# Hypoxanthine induces cholesterol accumulation and incites atherosclerosis in apolipoprotein E‐deficient mice and cells

**DOI:** 10.1111/jcmm.12916

**Published:** 2016-07-11

**Authors:** Hye‐Myung Ryu, You‐Jin Kim, Eun‐Joo Oh, Se‐Hyun Oh, Ji‐Young Choi, Jang‐Hee Cho, Chan‐Duck Kim, Sun‐Hee Park, Yong‐Lim Kim

**Affiliations:** ^1^ Division of Nephrology and Department of Internal MedicineKyungpook National University HospitalDaeguKorea; ^2^ BK21 Plus KNU Biomedical Convergence ProgramDepartment of Biomedical ScienceKyungpook National UniversityDaeguKorea; ^3^ Cell and Matrix Research InstituteKyungpook National UniversityDaeguKorea

**Keywords:** APOE, atherosclerosis, cholesterol, hypoxanthine, ROS

## Abstract

Reactive oxygen species (ROS) generation during purine metabolism is associated with xanthine oxidase and uric acid. However, the direct effect of hypoxanthine on ROS generation and atherosclerosis has not been evaluated. Smoking and heavy drinking are associated with elevated levels of hypoxanthine. In this study, we investigated the role of hypoxanthine on cholesterol synthesis and atherosclerosis development, particularly in apolipoprotein E (APOE)‐deficient mice. The effect of hypoxanthine on the regulation of cholesterol synthesis and atherosclerosis were evaluated in *Apoe* knockout (KO) mice and cultured HepG2 cells. Hypoxanthine markedly increased serum cholesterol levels and the atherosclerotic plaque area in *Apoe* KO mice. In HepG2 cells, hypoxanthine increased intracellular ROS production. Hypoxanthine increased cholesterol accumulation and decreased APOE and ATP‐binding cassette transporter A1 (ABCA1) mRNA and protein expression in HepG2 cells. Furthermore, H_2_O_2_ also increased cholesterol accumulation and decreased APOE and ABCA1 expression. This effect was partially reversible by treatment with the antioxidant N‐acetyl cysteine and allopurinol. Hypoxanthine and APOE knockdown using APOE‐siRNA synergistically induced cholesterol accumulation and reduced APOE and ABCA1 expression. Hypoxanthine induces cholesterol accumulation in hepatic cells through alterations in enzymes that control lipid transport and induces atherosclerosis in APOE‐deficient cells and mice. These effects are partially mediated through ROS produced in response to hypoxanthine.

## Introduction

Hypoxanthine, xanthine and uric acid are the products of the purine catabolism pathway 1. The generation of reactive oxygen species (ROS) during purine metabolism is well known 2. Xanthine oxidase and uric acid have been intensively studied. However, the direct effect of hypoxanthine on ROS generation and atherosclerosis has not been evaluated. Abnormal purine metabolism and purine overproduction is related to Lesch–Nyhan syndrome by purine salvage enzyme–guanine phosphoribosyltransferase (HGPRT) deficiency 3. Hypoxanthine acts as a substrate for xanthine oxidase and is associated with ROS, which contribute to vascular dysfunction and/or atherosclerosis 4, 5, 6. Cigarette smoking lowers HGPRT activity and may increase the plasma hypoxanthine level 7. Alcohol increases plasma concentrations of hypoxanthine 8. In addition, plasma hypoxanthine levels increase in patients on haemodialysis, who present high risks for cardiovascular diseases 9. Cigarette smoking, in addition to increasing the risk of cardiovascular diseases, is known to increase total cholesterol (TC), triglycerides (TG) and low‐density lipoprotein (LDL). Alterations of the enzymes that control lipid transport may be a key underlying mechanism 10. Although daily low to moderate alcohol intake is inversely related to cardiovascular diseases, alcohol itself induces oxidative stress and decreases apolipoprotein B levels. Heavy drinking may increase the risk of heart disease, hypertension and stroke 11. Hypoxanthine could be associated with a high risk of cardiovascular dysfunction and lipogenesis disorder in smoking or heavy‐drinking populations and in patients on haemodialysis. However, there is no direct evidence to support this association in the literature.

In this study we investigated (*i*) the effects of hypoxanthine on hyperlipidaemia and the formation of aortic atherosclerotic plaque *in vivo* using *Apoe* knockout (KO) and C57BL/6 mice; (*ii*) the effects of hypoxanthine on the regulation of cholesterol, enzymes that control lipid transport and lipogenesis‐related genes in HepG2 cells; (*iii*) the synergistic effects of hypoxanthine and apolipoprotein E (APOE) knockdown using siRNA on the regulation of cholesterol and enzymes that control lipid transport; (*iv*) the direct effect of hypoxanthine on ROS generation and its relationship with cholesterol metabolism in HepG2 cells.

## Materials and methods

### Animals, diets and specimen collections

Seven‐week‐old male Apoe KO mice were obtained from the Jackson Laboratories (Bar Harbor, ME, USA). C57BL/6 wild‐type (WT) mice were obtained from Samtako (O‐san, Korea). Animal experiments were approved by the Animal Care and Use Committee of Kyungpook National University and conformed to the Guide for the Care and Use of Laboratory Animals (NIH, Bethesda, MD, USA). Animals were fed with a high‐fat, high‐cholesterol diet (Research Diets, diet D12336; New Brunswick, NJ, USA) containing 16.0% fat, 1.25% cholesterol and 0.5% sodium cholic acid for 12 weeks. Fourteen WT mice were randomized into the hypoxanthine‐non‐treated (WT, *n* = 6) and ‐treated groups (WT‐Hx, *n* = 8). Another 14 Apoe KO mice were randomized into the hypoxanthine‐non‐treated (Apoe KO, *n* = 6) and ‐treated groups (Apoe KO‐Hx, *n* = 8). After 4 weeks of high‐fat diet feeding, hypoxanthine was given intraperitoneally (200 mg/kg) daily for 8 weeks. At the end of *in vivo* experiments (12 weeks), the animals were killed under anaesthesia breathing 1.5% isofluorane on a mask, the aortae were excised and analysed histologically. Liver was snap frozen in liquid nitrogen and stored in −80°C for lipid measurements. The blood was harvested for serum chemistry tests.

### Serum chemistry

At the end of the experimental period, blood samples from each mouse were collected into tubes by cardiac puncture. The blood was sampled from the heart of the mouse and was prepared in ethylenediaminetetraacetic acid‐free bottles to prevent coagulation. The lipid TC and TG levels in the serum were analysed by Samkwang Laboratory (Daegu, Korea).

### Assessment of atherosclerotic plaques in the aorta

After perfusion with cold PBS followed by perfusion of cold‐buffered formalin, aortas were excised from the thoracic cavity and heart. The aorta was then opened lengthwise and flat‐mounted on wax boards, and stained with 0.5% Sudan IV solution. The *en face* preparations were digitally photographed, then quantified using Image J (NIH), and the percentage of plaque coverage was calculated.

### HepG2 cell culture and treatment

Human HepG2 hepatic carcinoma cells were purchased from the American Type Culture Collection (ATCC; Manassas, VA, USA). HepG2 cells were cultured at 37°C in 5% CO_2_ in DMEM (Gibco‐BRL, Gaithersburg, MD, USA) supplemented with 10% foetal bovine serum (Gibco‐BRL), 100 μg/ml penicillin and 10 μg/ml streptomycin (Gibco‐BRL). HepG2 cells were seeded in wells of a standard 12‐well or 6‐well plate. Growth medium was changed every 3–4 days or as required. HepG2 cells were incubated in 10% DMEM in the absence or presence of various concentrations of hypoxanthine (1 and 2.5 mM), H_2_O_2_ (0, 100, 250, 500 and 1000 μM), 5 mM *N*‐acetylcysteine (NAC; Sigma‐Aldrich, Saint Louis, MO, USA) and 100 μM allopurinol (Sigma‐Aldrich) for 24 hrs at 37°C.

### Determination of TC and cholesteryl ester

The TC and free cholesterol content of the liver and HepG2 cell were measured colorimetrically using a Cholesterol assay kit according to the manufacturer's protocol (ab65359; Abcam, Cambridge, UK). Ten milligrams of liver tissue was extracted with 200 μl of chloroform: Isopropanol: NP‐40 (7:11:0.1) in a glass homogenizer. The extract was centrifuged at 15,000 × *g* for 10 min and the liquid phase was transferred to a new tube and air‐dried at 50°C. The dried samples were dissolved in 200 μl of 1× assay diluent following the protocol provided by the manufacturer. HepG2 cells were incubated in the absence or presence of hypoxanthine or H_2_O_2_ for 24 hrs at 37°C. Cells were washed in ice‐cold saline. Cholesterol and cholesteryl ester contents in cells were measured using cholesterol assay buffer after extracted with chloroform: Isopropanol: NP‐40 (7:11:0.1). All samples were incubated with the cholesterol assay reaction buffer at 37*°*C for 60 min. The absorbance was measured at 570 nm.

### Intracellular ROS measurement

Intracellular ROS generation was measured using 2′,7′‐dichlorofluorescein diacetate (DCFH‐DA). HepG2 cells were seeded at a density of 1 × 10^4^ cells/well in a black 96‐well plate. 2′,7′‐dichlorofluorescein diacetate is hydrolysed by esterases to DCFH, which is trapped within the cell. This non‐fluorescent molecule is then oxidized to fluorescent dichlorofluorescein (DCF) by the action of cellular oxidants. In the NAC pre‐treatment groups, the medium containing 5 mM NAC was added and the cells were pre‐incubated for 2 hrs. Once pre‐treated, the cells were washed twice with PBS and incubated with a solution of 50 μM DCFH‐DA in serum‐free medium for 30 min. This was followed by washing the cells twice with PBS. The cells were then incubated with either hypoxanthine/H_2_O_2_ or medium for 2 hrs. Intracellular ROS levels were determined using a fluorescence microscope.

### Determination of hydrogen peroxide

The levels of H_2_O_2_ were measured using the Amplex Red Hydrogen Peroxide Assay Kit according to the manufacturer's protocol (Molecular Probes, Invitrogen, Eugene, OR, USA). In the presence of horseradish peroxidase, Amplex red reacts with H_2_O_2_ in a 1:1 stoichiometry to produce the red‐fluorescent oxidation product resorufin. Experiments were done in a 96‐well plate in a total volume of 0.1 ml. The fluorescence was then determined at 545 nm excitation and 590 nm emission, using a microplate reader (FLUOstar; BMG Labtechnologies, Durham, NC, USA).

### Determination of cell viability

HepG2 cells (1.0 × 10^4^), cultured in a 96‐well plate in DMEM, were treated with H_2_O_2_ (0, 100, 500 and 1000 μM) for 2 or 24 hrs. After medium exchange, HepG2 cells were cultured for 24 hrs and treated with 500 μg/ml 3‐(4,5‐dimethylthiazol‐2yl)‐2,5‐diphenyltetrazolium bromide (MTT) for 4 hrs. After melting the formazan by addition of 200 μl dimethyl sulfoxide solution (Amresco, Solon, OH, USA), the absorbance was measured at 570 nm using a microplate reader (Model 550; Bio‐Rad, Hercules, CA, USA).

### Transfection of HepG2 with APOE‐siRNA

Human APOE‐siRNA and non‐targeting siRNA, negative control, were purchased from Dharmacon (Chicago, IL, USA) and used at 20 nM. Opti‐MEM^™^ transfection medium and Lipofectamine^™^ (both from Invitrogen, Paisley, UK) were used for transfection. HepG2 cells were seeded one day prior to transfection and cultured so that they were 40–50% confluent on the following day. RNAi duplexes for APOE were mixed with Lipofectamine to form a transfection complex that was added to the plated cells. After 4 hrs of incubation, the medium was replaced with DMEM and cells were incubated for 24 hrs. Real‐time reverse transcription‐polymerase chain reaction (RT‐PCR) and western blot analysis were performed.

### Western blotting

Western blotting was performed as described previously 12. Primary antibodies against APOE (1:2000; Abcam), ATP‐binding cassette, sub‐family A member 1 (ABCA1, 1:2000; Abcam), 3‐hydroxy‐3‐methylglutaryl coenzyme A reductase (HMGCR, 1:5000; Abcam), LDL receptor (LDLR, 1:2000; Abcam) and β‐actin (1:10,000; Sigma‐Aldrich) were used. A horseradish‐peroxidase‐conjugated polyclonal goat anti‐rabbit immunoglobulin or goat anti‐mouse immunoglobulin (Dako, Glostrup, Denmark) was used as the secondary antibody for western blotting. Positive immunoreactive bands were quantified by densitometry and compared with human β‐actin expression.

### RNA isolation and quantitative real‐time RT‐PCR analysis

Total RNA was isolated from cell lysates using the TRIreagent (Molecular Research Center Inc., Cincinnati, OH, USA) according to the provider's instructions. One microgram of total RNA was reverse transcribed using the Prime Script cDNA Synthesis kit (Takara Shuzo Co., Otsu, Shiga, Japan). Quantitative real‐time RT‐PCR was performed using gene‐specific primers and the SYBR green PCR Master Mix (Applied Biosystems, Foster City, CA, USA) in PRISM 7700 Sequence Detection System (Applied Biosystems) as previously described 12. All samples were quantified using the comparative Ct method for relative quantification of gene expression. The expression of genes of interest was normalized to that of β‐actin. The primer sets used in this study are listed in Table S1.

### Statistical analysis

The data shown are the mean ± S.E.M. All experiments were repeated at least three independent times. All data were tested for normality using the Shapiro–Wilks normality test. Significance of difference between groups was analysed by performing one‐way anova followed by Dunett's *post hoc* test or Bonferroni *post hoc* test. Non‐normally distributed data were analysed by Kruskal–Wallis test. A *P*‐value less than 0.05 was considered statistically significant. Statistical analysis was performed with GraphPad Prism 5 (Graph Pad, La Jolla, CA, USA).

## Results

### Hypoxanthine induces hypercholesterolaemia and accelerates atherosclerotic plaque formation in Apoe KO mice

**Figure 1:**
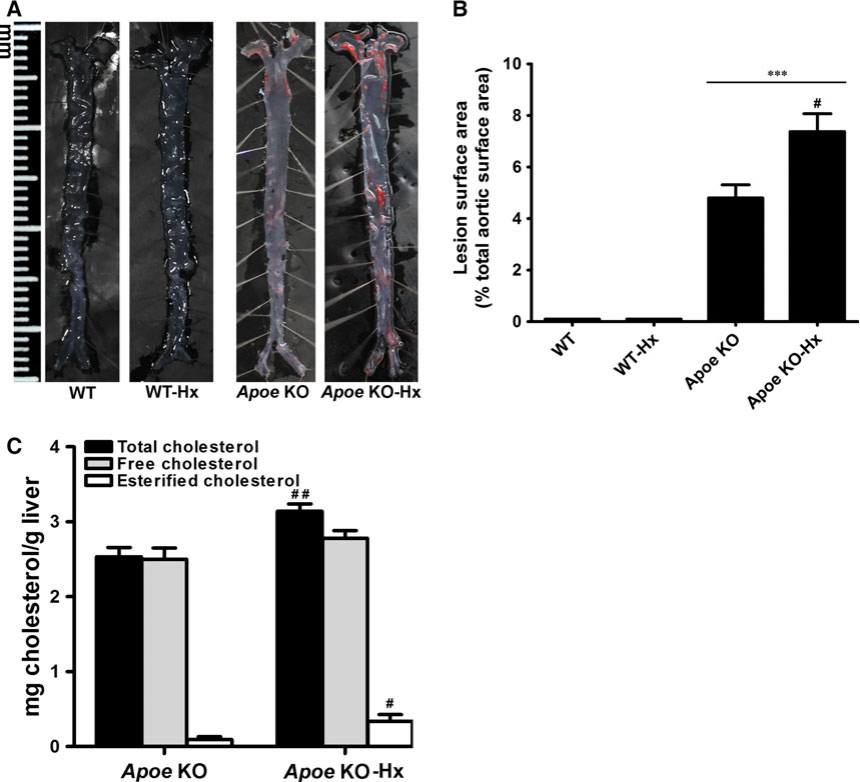
Hypoxanthine increases atherosclerotic plaque formation in *Apoe* KO mice. (**A**) Representative photographs of *en face* of aortic sinus from C57BL/6 (WT) and *Apoe* KO mice. (**B**) Quantitative analysis of *en face* area of the aorta from WT, WT‐Hx, *Apoe* KO and *Apoe* KO‐Hx mice. (**C**) Hepatic total cholesterol and cholesteryl ester levels were assayed in liver. Data are presented as mean ± S.E.M. ***P < 0.001 *versus* WT, ^#^ *P* < 0.05, ^#^^#^ *P* < 0.01 *versus Apoe* KO. Hx, hypoxanthine.

To address whether hypoxanthine can alter the development of atherosclerosis, the effects of hypoxanthine on lipid profiles and plaque formation were assessed. Seven‐week‐old male *Apoe* KO mice and WT mice were fed with a high‐fat, high‐cholesterol diet for 12 weeks. After 4 weeks of high‐fat diet feeding, hypoxanthine was then administered for 8 weeks (200 mg/kg/day). At the end of *in vivo* experiments (12 weeks), hypoxanthine did not affect bodyweight in both WT and *Apoe* KO mice. Interestingly, hypoxanthine treatment significantly increased serum TC level in *Apoe* KO mice compared to untreated *Apoe* KO control mice (1578 ± 172 *versus* 370 ± 17; *P* < 0.05; Table 1). However, there was no difference between the hypoxanthine and untreated WT mice. Analysis of the *en face* aorta demonstrated a marked induction of atherosclerotic plaque formation in the hypoxanthine‐treated compared to the untreated *Apoe* KO mice. Hypoxanthine treatment did not induce plaque formation in C57BL/6 mice (Fig. 1A and B). To examine whether atherosclerosis was accompanied by a biochemical increase in hepatic lipid levels, we measured TC, free cholesterol and cholesterol ester in the liver tissue. The hepatic TC and cholesterol ester levels significantly increased in hypoxanthine‐treated *Apoe* KO mice compared to untreated *Apoe* KO mice (Fig. 1C).

**Table 1:** Effect of hypoxanthine on serum lipids in *Apoe* KO and C57BL/6 mice

	WT (*n* = 6)	WT‐Hx (*n* = 8)	*Apoe* KO (*n* = 6)	*Apoe* KO‐Hx (*n* = 8)
Bodyweight	26.3 ± 1.2	26.0 ± 0.6	27.1 ± 0.8	26.8 ± 0.6
Total cholesterol (mg/dl)	144 ± 8	136 ± 3	370 ± 17*	1578 ± 172* ^,^ †
Triglyceride (mg/dl)	34 ± 1	36 ± 2	83 ± 12	150 ± 81

*P < 0.001 *versus* WT mice, ^†^ *P* < 0.001 *versus Apoe* KO mice. Data are expressed as mean ± S.E.M. Data were analysed with one‐way anova and Bonferroni *post hoc* test; WT, C57BL/6 wild type; Hx: hypoxanthine.

### Hypoxanthine induces ROS generation and cholesterol up‐regulation in HepG2 cells

**Figure 2:**
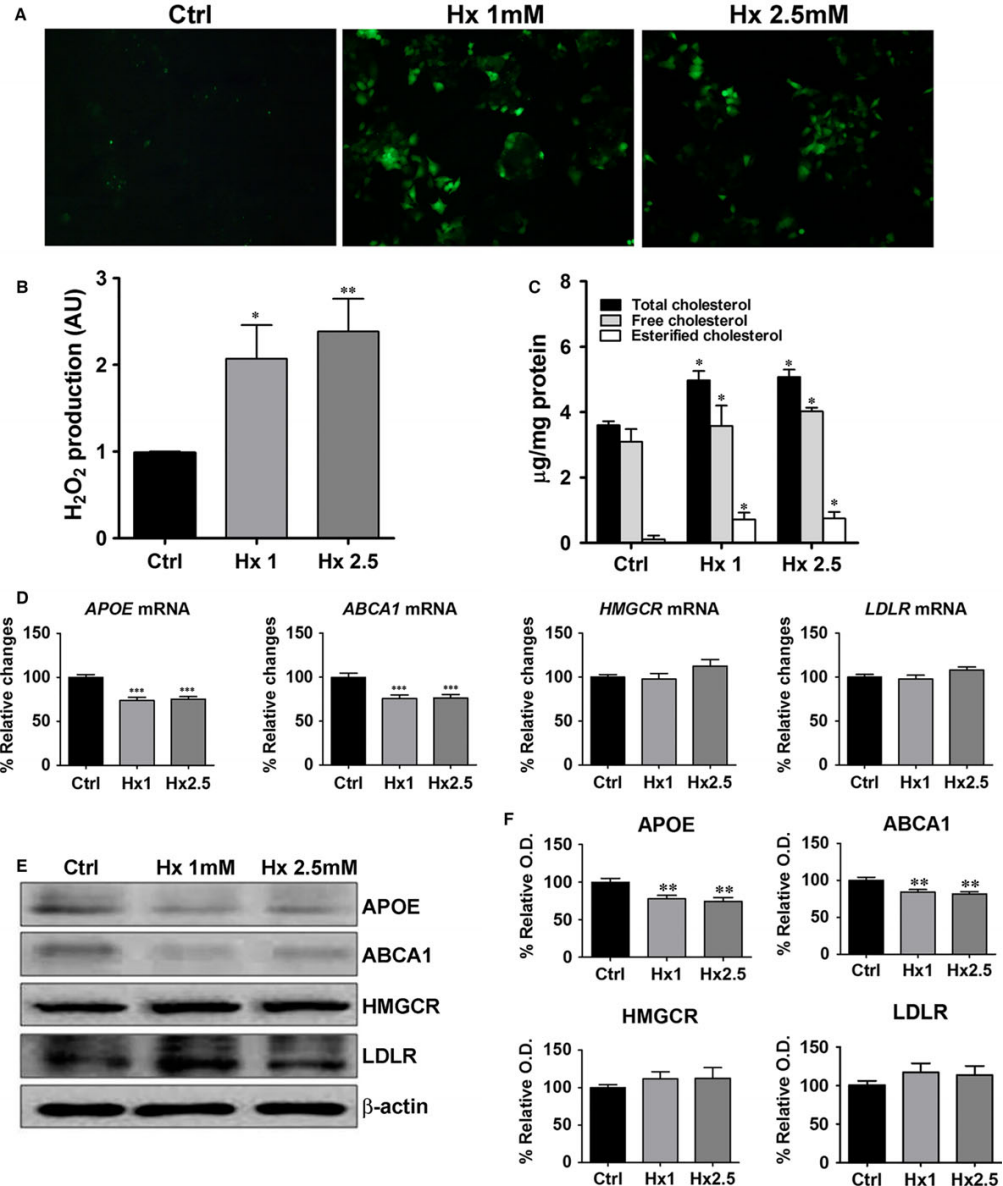
Hypoxanthine induces ROS generation and cholesterol accumulation in HepG2 cells by down‐regulation of reverse cholesterol transport gene. (**A**) HepG2 cells were treated with different concentrations of hypoxanthine (1, 2.5 mM) for 2 hrs and ROS levels were evaluated by the oxidation of H2DCF‐DA to DCF. Intracellular ROS levels were determined using a fluorescence microscope (magnification, 400×). (**B**) The levels of H_2_O_2_ were determined by the Amplex red assay. (**C**) HepG2 cells were treated with different concentrations of hypoxanthine (1, 2.5 mM) for 24 hrs and total cholesterol and cholesteryl ester levels were assayed in cell lysate. (**D**) HepG2 cells were treated with different concentrations of hypoxanthine (1, 2.5 mM) for 24 hrs. *APOE*,*ABCA1*,*HMGCR* and *LDLR* mRNA levels were determined by real‐time RT‐PCR. (**E**) Representative western blot for APOE, ABCA1, HMGCR, LDLR and β‐actin is shown. (**F**) Relative protein expression was determined using densitometry. Data are presented as mean ± S.E.M. *P < 0.05, **P < 0.01, ***P < 0.001 *versus* control (Ctrl). AU: arbitrary units.

The effects of hypoxanthine were investigated *in vitro*. To examine the potential mechanism involved in hypoxanthine‐induced cholesterol up‐regulation, HepG2 hepatic carcinoma cells were treated with hypoxanthine for 24 hrs. HepG2 cell viability was decreased in a dose‐ and time‐dependent manner (Fig. S1). Reactive oxygen species production and TC and cholesteryl ester levels in cell lysates were examined. Notably, the ROS‐dependent DCF green fluorescence intensity and the levels of H_2_O_2_ were increased after exposure to hypoxanthine (1 and 2.5 mM) for 2 h (Fig. 2A and B). After exposure to hypoxanthine (1 and 2.5 mM) for 24 hrs, TC and cholesteryl ester levels were significantly higher in hypoxanthine‐treated HepG2 cells (1 and 2.5 mM) than in control cells (Fig. 2C).

### Hypoxanthine down‐regulates genes involved in reverse cholesterol transport

To further clarify the mechanism of hypoxanthine‐induced cholesterol accumulation, the expression of lipogenesis‐related genes was examined. *APOE* and *ABCA1* genes play crucial roles in the formation of HDL 13. Hypoxanthine significantly decreased APOE and ABCA1 mRNA and protein levels. However, HMGCR and LDLR mRNA and protein levels were not affected by hypoxanthine (Fig. 2D–F).

### Effects of ROS on cholesterol accumulation in H_2_O_2_‐treated HepG2 cells

**Figure 3:**
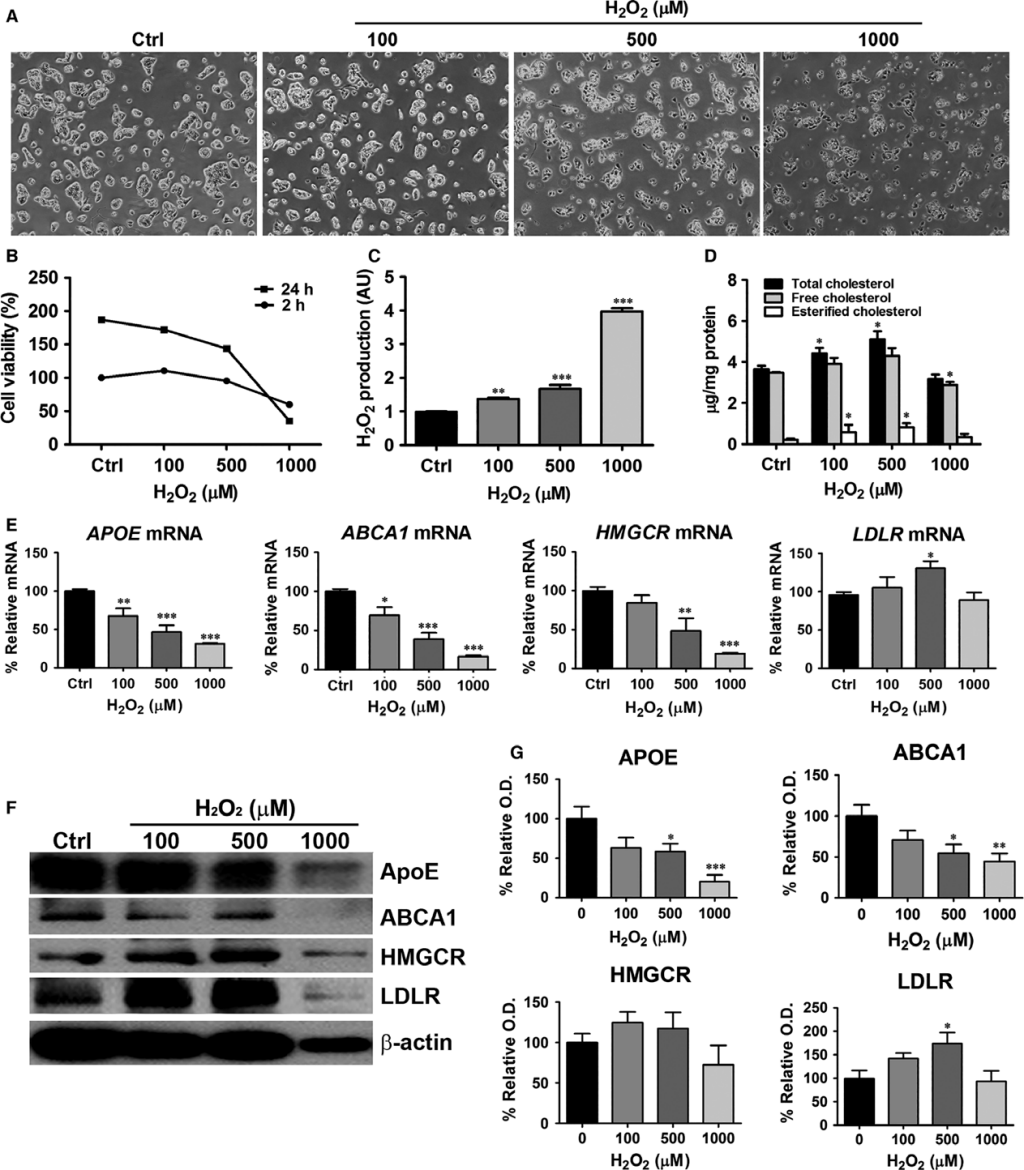
Effects of H_2_O_2_ on cholesterol accumulation and lipogenesis‐related genes in HepG2 cells. HepG2 cells were treated with H_2_O_2_ (100, 500 and 1000 μM) for 2 hrs (for Amplex red assay) or 24 hrs (for cholesterol quantification, real‐time RT‐PCR and western blot analysis). (**A**) Light micrographs (magnification 40×) show that H_2_O_2_ reduced cell viability. (**B**) Cell viability was determined using the MTT assay. (**C**) The levels of H_2_O_2_ were determined by the Amplex red assay. (**D**) Cellular total cholesterol and cholesteryl ester levels were determined from cell lysates. (**E**) *APOE*,*ABCA1*,*HMGCR* and *LDLR* mRNA levels were determined by real‐time RT‐PCR. (**F**) Representative western blot for APOE, ABCA1, HMGCR, LDLR and β‐actin is shown. (**G**) Relative protein expression was determined using densitometry. Data are presented as mean ± S.E.M. *P < 0.05, **P < 0.01 *versus* control, ***P < 0.001 *versus* control (Ctrl). AU: arbitrary units.

In the next series of experiments, to evaluate whether cholesterol up‐regulation is as a result of ROS induction, HepG2 cells were exposed to H_2_O_2_ (100, 500 and 1000 μM) for 2 and 24 hrs. Cell viability, ROS level and the level of cholesterol were assayed. After incubation with H_2_O_2_ for 2 and 24 hrs, cell viability was significantly decreased in the 500 and 1000 μM H_2_O_2_‐treated groups (Fig. 3A and B). After exposure to H_2_O_2_ for 2 hrs, the level of H_2_O_2_ significantly increased compared to that of control cells (Fig. 3C). After exposure to H_2_O_2_ for 24 hrs, TC and cholesteryl ester levels were significantly higher in H_2_O_2_ treated HepG2 cells than in control cells (Fig. 3D). However, at 1 mM H_2_O_2_, cholesterol levels decreased because of severe cytotoxic effects.

### Effects of ROS on lipogenesis‐related genes in H_2_O_2_‐treated HepG2 cells

To further evaluate the mechanism of H_2_O_2_‐induced cholesterol accumulation, the expression of lipogenesis‐related genes was examined. APOE and ABCA1 gene and protein expression decreased significantly after H_2_O_2_ treatment. However, LDLR mRNA and protein levels increased significantly after treatment with 500 μM H_2_O_2_. *HMGCR* mRNA decreased in dose‐dependent manner in the presence of H_2_O_2_, whereas protein levels were not significantly affected compared to that of control cells (Fig. 3E–G). At 1 mM H_2_O_2_ concentration, morphology of HepG2 cells was changed and cell viability decreased (Fig. 3A and B). The density of the actin band also decreased at 1 mM H_2_O_2_ (Fig. 3F). Therefore, the concentration of 500 μM H_2_O_2_ was used to examine the H_2_O_2_‐induced oxidative effects on HepG2 cells.

### Effects of NAC on ROS generation and TC up‐regulation

**Figure 4:**
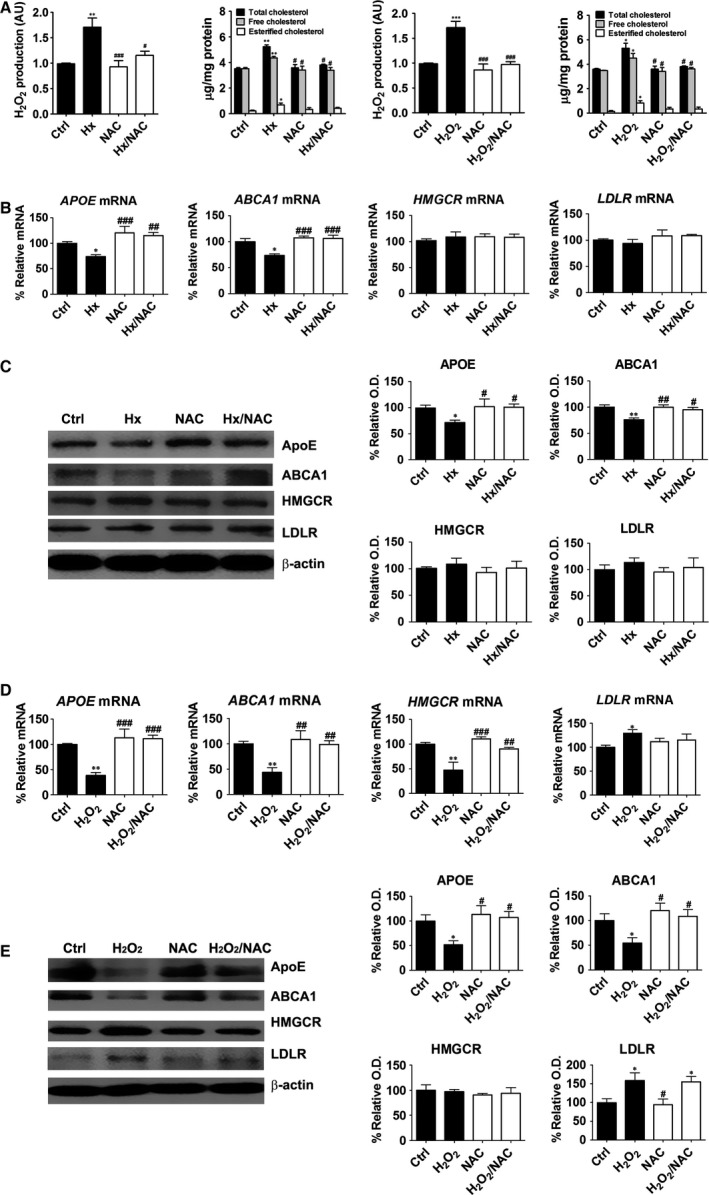
Effects of NAC on cholesterol levels and lipogenesis‐related genes in hypoxanthine‐ or H_2_O_2_‐treated HepG2 cells. Cells were treated with hypoxanthine (1 mM) or H_2_O_2_ (500 μM) for 2 hrs (for Amplex red assay) or 24 hrs (for cholesterol quantification) following a 2 hrs pre‐incubation with 5 mM NAC. (**A**) The levels of H_2_O_2_ were determined by the Amplex red assay and total cholesterol and cholesteryl ester levels were assayed in cell lysates. (**B**) Cells were treated with 1 mM hypoxanthine for 24 hrs, following a 2 hrs pre‐incubation with 5 mM NAC. *APOE*,*ABCA1*,*HMGCR* and *LDLR* mRNA levels were determined by real‐time RT‐PCR. (**C**) APOE, ABCA1, HMGCR, LDLR and β‐actin protein levels were measured by western blot analysis. (**D**) Cells were treated with 500 μM H_2_O_2_ for 24 hrs, following a 2 hrs pre‐incubation with 5 mM NAC. *APOE*,*ABCA1*,*HMGCR* and *LDLR* mRNA levels were determined by real‐time RT‐PCR. (**E**) APOE, ABCA1, HMGCR, LDLR and β‐actin protein levels were measured by western blot analysis. Relative protein expression was determined using densitometry. Data are presented as the mean ± S.E.M. *P < 0.05, **P < 0.01, ***P < 0.001 *versus* control (Ctrl). #*P* < 0.05, ##*P* < 0.01, ###*P* < 0.001 *versus* hypoxanthine (Hx). AU: arbitrary units.

Next, we examined the effect of antioxidants on hypoxanthine‐ and H_2_O_2_‐induced cholesterol accumulation. HepG2 cells were pre‐treated with NAC at 5 mM for 2 hrs, followed by hypoxanthine (1 mM) or H_2_O_2_ (500 μM) treatment. As shown in Figure 4, H_2_O_2_ level was decreased after treatment with 5 mM NAC and remained similar to the level observed in control cells. Total cholesterol level was up‐regulated in HepG2 cells exposed to hypoxanthine and H_2_O_2_, whereas NAC ameliorated cholesterol accumulation. These results indicate that NAC, an ROS scavenger, could inhibit ROS‐induced cholesterol accumulation (Fig. 4A).

### Effects of NAC on lipogenesis‐related genes in hypoxanthine‐ or H_2_O_2_‐treated HepG2 cells

To evaluate whether hypoxanthine‐induced cholesterol up‐regulation was mediated through ROS, we examined the effect of antioxidants on lipogenesis‐related gene levels. *N*‐acetylcysteine treatment abolished the inhibitory effect of hypoxanthine and H_2_O_2_ on APOE and ABCA1 expression. However, HMGCR and LDLR mRNA and protein levels were not affected by hypoxanthine and NAC treatment. Some differences in HMGCR and LDLR expression were observed after H_2_O_2_ treatment (Fig. 4B and C). *HMGCR* transcripts were significantly decreased by H_2_O_2_ treatment and NAC abolished the effect of H_2_O_2_ on *HMGCR* expression. HMGCR protein level was not significantly affected compared to that of control cells. LDLR mRNA and protein levels were significantly increased by H_2_O_2_ treatment, but were not affected by NAC treatment (Fig. 4D and E).

### Effects of allopurinol on ROS generation and cholesterol up‐regulation

**Figure 5:**
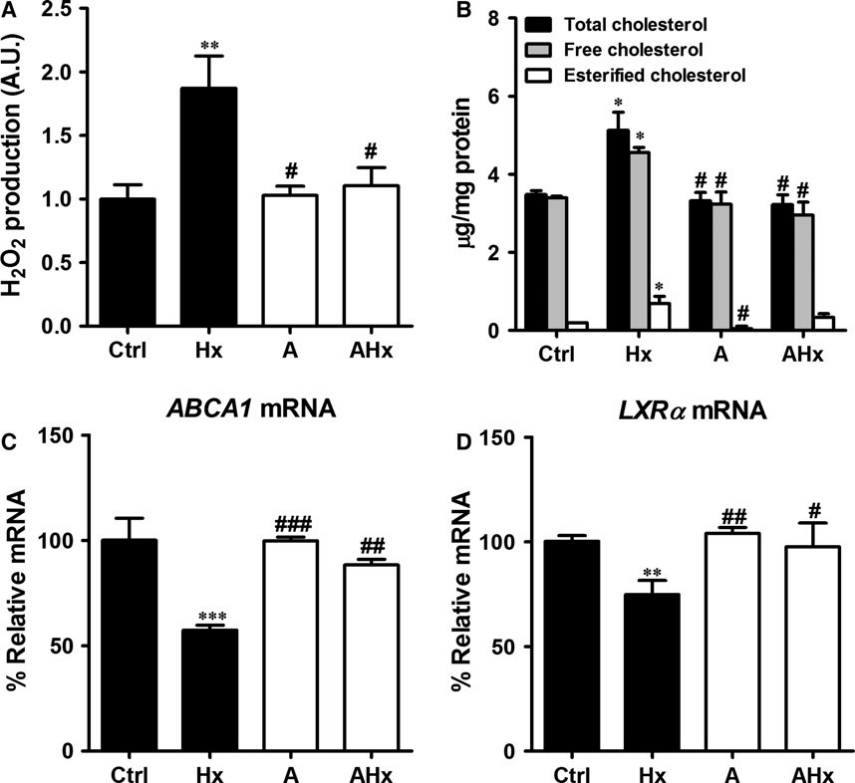
Effects of allopurinol on cholesterol levels and lipogenesis‐related genes in hypoxanthine‐treated HepG2 cells. Cells were treated with hypoxanthine (1 mM) and allopurinol (100 μM) for 2 hrs (for Amplex red assay) or 24 hrs (for cholesterol quantification and real‐time RT‐PCR). (**A**) The levels of H_2_O_2_ were determined by the Amplex red assay. (**B**) Cellular total cholesterol and cholesteryl ester levels were determined from cell lysates. (**C**) *ABCA1* and (**D**) *LXR‐a* mRNA levels were determined by real‐time RT‐PCR. Data are presented as mean ± S.E.M. *P < 0.05, **P < 0.01, ***P < 0.001 *versus* control (Ctrl). ^#^ *P* < 0.05, ^#^^#^ *P* < 0.01, ^#^^#^^#^ *P* < 0.001 *versus* hypoxanthine (Hx). A: allopurinol; AU: arbitrary units.

To investigate the effects of specific xanthine oxidase inhibitor, allopurinol was used. Reactive oxygen species production and TC levels were evaluated by co‐treatement with allopurinol (100 μM) and hypoxanthine (1 mM) for 2 and 24 hrs. Allopurinol treatment significantly reduced ROS production and cholesterol accumulation (Fig. 5A and B). Next, we examined the mRNA expression of ABCA1 and liver X receptor alpha (LXR‐α) genes in HepG2 cells (Fig. 5C and D). In allopurinol‐treated group, ABCA1 and LXR‐α mRNA expressions were significantly increased compared with hypoxanthine‐treated group.

### Effects of hypoxanthine on cholesterol accumulation and lipogenesis‐related genes in the APOE‐deficient state

**Figure 6:**
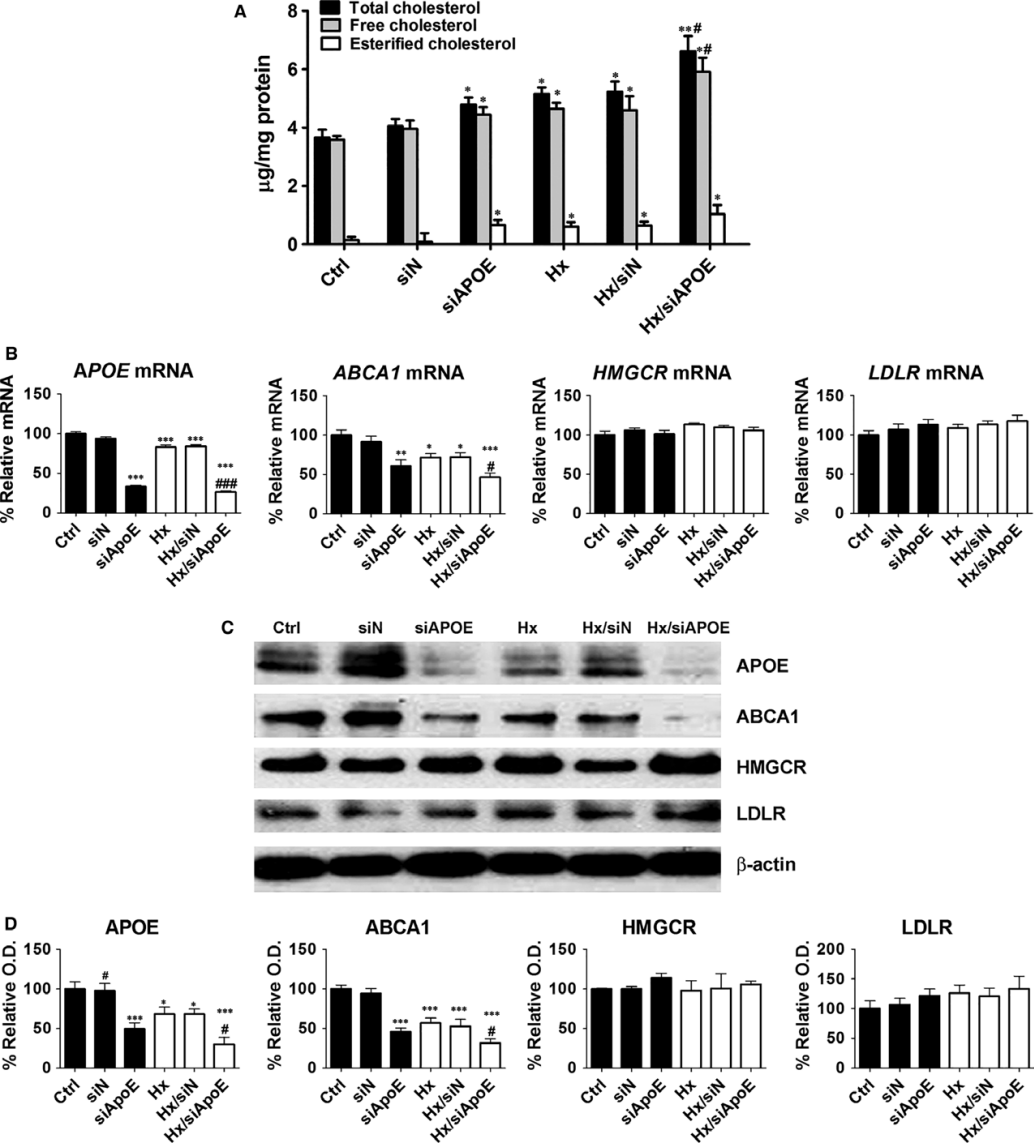
Effects of hypoxanthine on cholesterol levels and lipogenesis‐related genes in APOE‐deficient HepG2 cells. (**A**) HepG2 cells were transfected with APOE‐siRNA (siAPOE) or non‐target‐siRNA (siN) for 24 hrs and then treated with hypoxanthine (1 mM) for 24 hrs. Total cholesterol and cholesteryl ester levels were assayed in cell lysates. (**B**) *APOE*,*ABCA1*,*HMGCR* and *LDLR* mRNA levels were determined by real‐time RT‐PCR. (**C**) Representative western blot for APOE, ABCA1, HMGCR, LDLR and β‐actin is shown. (**D**) Relative protein expression was determined using densitometry. Data are presented as the mean ± S.E.M. *P < 0.05, **P < 0.01, ***P < 0.001 *versus* control (Ctrl). ^#^ *P* < 0.05, ^#^^#^^#^ *P* < 0.001 *versus* hypoxanthine (Hx).

To evaluate the effects of hypoxanthine on cholesterol accumulation in APOE‐deficient cells, human APOE‐siRNA (siAPOE; 20 nM) or non‐targeting siRNA (siN; 20 nM) was transfected into HepG2 cells. Twenty‐four hours after siAPOE or siN transfection, HepG2 cells were treated with 1 mM hypoxanthine or left untreated for 24 hrs. APOE knockdown increased cholesterol accumulation. Co‐treatment with hypoxanthine and siAPOE synergistically induced cholesterol accumulation (Fig. 6A). Apolipoprotein E knockdown diminished *APOE* and *ABCA1* transcript levels. Moreover, hypoxanthine and siAPOE synergistically decreased APOE and ABCA1 mRNA and protein levels. 3‐hydroxy‐3‐methylglutaryl coenzyme A reductase and LDLR mRNA and protein levels were not affected by hypoxanthine and siAPOE (Fig. 6B–D).

## Discussion

This study shows that hypoxanthine induces cholesterol accumulation in hepatic cells through alterations in the enzymes that control lipid transport and the development of atherosclerosis in APOE‐deficient cells and mice. The direct relationship between hypoxanthine and cholesterol accumulation or atherosclerosis has not been previously reported and is a novel finding.

Hypoxanthine is a naturally occurring purine derivative in purine metabolism, adenosine is deaminated into inosine. Inosine is then converted to hypoxanthine, which is converted to xanthine and uric acid by xanthine oxidase 1. Uric acid is well‐known risk factor of cardiovascular 14, 15. However, there is a controversy for the influence of hyperuricemia on atherosclerosis. The Atherosclerosis Risk in Communities Study (ARIC Study) reported that uric acid *per se* may not be a risk factor for atherosclerosis 16. In this study, we assessed the serum uric acid level. The uric acid level significantly increased in *Apoe* KO mice compared to C57BL/6 mice (WT). However, there was no difference of uric acid level between the hypoxanthine‐treated and untreated *Apoe* KO groups (Fig. S2). Xanthine oxidase is a critical source of ROS, which contribute to vascular dysfunction observed in ischaemia/reperfusion injury 5.

Many *in vitro* and animal studies implicated inflammation and oxidative stress in purine metabolism and vascular injury. To this end, xanthine oxidase and uric acid received massive attention, but hypoxanthine did not 17.

Although serum/urine hypoxanthine measurement has been reported to provide valuable information for the diagnosis and medical management of certain metabolic disorders such as gout and renal failure 18, 19, its direct impact on atherosclerosis and/or lipogenesis has not been evaluated. However, the association between hypoxanthine and vascular injury has been established. Hypoxanthine increases in ischaemia/reperfusion injury of the myocardium and renal graft and is associated with ROS 4, 6.

Common lifestyle factors such as cigarette smoking and alcohol drinking are associated with increased hypoxanthine. Cigarette smoking lowers HGPRT activity, which may increase hypoxanthine levels as observed in Lesch–Nyhan syndrome 7. Ethanol also increases hypoxanthine plasma concentration and urinary excretion 8. In addition, hypoxanthine increases in haemodialysis without an increase in serum xanthine oxidase activity and uric acid levels 9. This study directly demonstrates that hypoxanthine induces cholesterol accumulation and accelerates atherosclerosis. This direct effect of hypoxanthine may contribute to a high risk of cardiovascular diseases in smoking or heavy‐drinking populations and in patients on haemodialysis. Furthermore, hypoxanthine level can be used as a biomarker of oxidative stress to predict cardiovascular diseases in these situations.

To investigate whether hypoxanthine can alter the development of atherosclerosis *in vivo*, the *Apoe* KO mouse model, which is a well‐known atherosclerosis animal model, and C57BL/6 mice were used. Hypoxanthine treatment did not induce plaque formation and TC increase in C57BL/6 mice, but did in *Apoe* KO mice. The effect of hypoxanthine on lipid and atherosclerosis seems to be accelerated in *Apoe*‐deficient mice because APOE triggers clearance of chylomicron, very LDL and LDL remnants and has an antioxidant effect. The antioxidant capacity of APOE is well known 20. Plasma lipoproteins in *Apoe*‐deficient mice are susceptible to oxidative stress 21. In humans, APOE deficiency or its abnormalities is associated with a series of pathological conditions, including hyperlipidaemia, atherosclerosis, Alzheimer's disease and shorter life span 22, 23, 24, 25. In peripheral tissues, APOE primarily mediates cholesterol metabolism in an isoform‐dependent manner 26. Allele frequencies of the human *APOE* gene are variable around the world. The frequency of ε3 (APOE3) is the highest, followed by ε4 (APOE4) and ε2 (APOE2) 27. APOE3 seems to be the normal isoform in all known functions, while APOE4 and APOE2 can each be dysfunctional. APOE4 is associated with increased cholesterol levels and heart disease. APOE4 carrier frequencies can vary from 5% to nearly 50% 27, 28. The antioxidative activity of APOE is also isoform‐dependent. APOE4 is the least effective 29. APOE4 and smoking synergistically increase the odds of carotid atherosclerosis 30. APOE4 carriers present a dysfunctional APOE status, which may be represented in the APOE‐deficient model used in our study. The impact of hypoxanthine on hypercholesterolaemia and atherosclerosis in human APOE4 carriers needs to be further evaluated.

Hypercholesterolaemia contributes to early atherosclerosis, mainly by inducing macrophage‐derived foam cell formation under conditions such as oxidative stress and dysfunction of cholesterol transportation in and out of the vessel wall 31. The liver is a major site for lipid homeostasis. Therefore, we examined changes in TC levels and expression of lipogenesis‐related genes after hypoxanthine treatment in human HepG2 cells. In this study, hypoxanthine treatment increased ROS generation and TC accumulation in HepG2 cells. In addition, cholesterol transport genes (*APOE* and *ABCA1*) were down‐regulated. HMGCR and LDLR transcripts and protein levels were not affected by hypoxanthine. H_2_O_2_ treatment not only showed similar effects, but also induced LDLR up‐regulation. NAC abrogated hypoxanthine's inhibitory effect on APOE and ABCA1 mRNA and protein expression and ameliorated cholesterol accumulation. These results indicate that hypoxanthine is critically involved in lipid accumulation by regulating APOE and ABCA1 expression in HepG2 cells, partially through ROS. However, as observed for LDLR expression, ROS‐independent pathways may also play a role.

One of the critical roles of APOE is to promote cholesterol efflux from macrophages 32, 33. APOE promotes macrophage cholesterol efflux through the ABCA‐1 and ABCG‐1 cell surface transporters, which facilitate the efflux of phospholipids and cholesterol onto lipid‐poor apolipoproteins, thereby initiating the formation of HDL particles 13. ABCA1 is one of the ABC superfamily members. ABCA1 transporter facilitates cellular phospholipids efflux and cholesterol to acceptors such as APOA‐I and APOE, which stabilize HDL particles. Transgenic mice that express a high level of ABCA1 present an increase in cholesterol efflux in macrophages and exhibit a low incidence of developing atherosclerosis. Selective inactivation of macrophage ABCA1 in mice results in a substantial increase in atherosclerosis 34, 35. Accordingly, overexpression of hepatic ABCA1 raises HDL cholesterol levels 36. Hepatic ABCA1 mRNA and protein levels are down‐regulated by hypoxanthine treatment in our study, which is one of main mechanisms involved in cholesterol accumulation. Allopurinol, a xanthine oxidase inhibitor, is known to protect against cardiovascular disease by reducing the formation of superoxide anion of xanthine oxidase and by directly scavenging free radicals and chelating non‐protein‐bound iron 37. Although several studies report that allopurinol treatment increase the concentration of hypoxanthine in patient 38, 39, the hypoxanthine level in HepG2 cell was not changed by allopurinol in our study (data not shown). Recently, Kushiyama *et al*. reported that allopurinol inhibits lipid accumulation in macrophage and inhibition of XO activity might be useful to preventing atherosclerosis 40. To investigate xanthine oxidase‐mediated ROS production, allopurinol was used in this study. The down‐regulation of ABCA1 by hypoxanthine was attenuated by allopurinol. Next, we investigated whether the ABCA1 is associated with the LXR‐α pathway. The LXRs are nuclear receptors that are activated by endogenous oxysterols, oxidized derivatives of cholesterol. The LXR‐α is associated with the expression of several LXR‐α target genes involved in cholesterol metabolism (apoE, ABCA1 and ABCG1) 41. Liver X receptor‐α mRNA was decreased in hypoxantine‐treated group and allopurinol treatment attenuated the down‐regulation of LXR‐α.

To better understand the factors that contribute to the effect of APOE deficiency on hepatic cholesterol metabolism, APOE‐siRNA was transfected into HepG2 cells. APOE knockdown increased cholesterol accumulation. Co‐treatment with hypoxanthine and APOE‐siRNA synergistically induced cholesterol accumulation by *APOE* and *ABCA1* down‐regulation. This finding is in agreement with one report describing an APOE‐mediated ABCA1‐dependent cellular cholesterol efflux. Joyce *et al*. reported that the absence of APOE in the *Apoe* KO mice would limit the effectiveness of ABCA1 overexpression in reducing atherosclerosis. ABCA1 overexpression in C57BL/6 mice on a high‐cholesterol diet results in an atheroprotective lipoprotein profile and decreased atherosclerosis, but ABCA1 overexpression in *Apoe* KO mice led to increased atherosclerosis 42.

**Figure 7:**
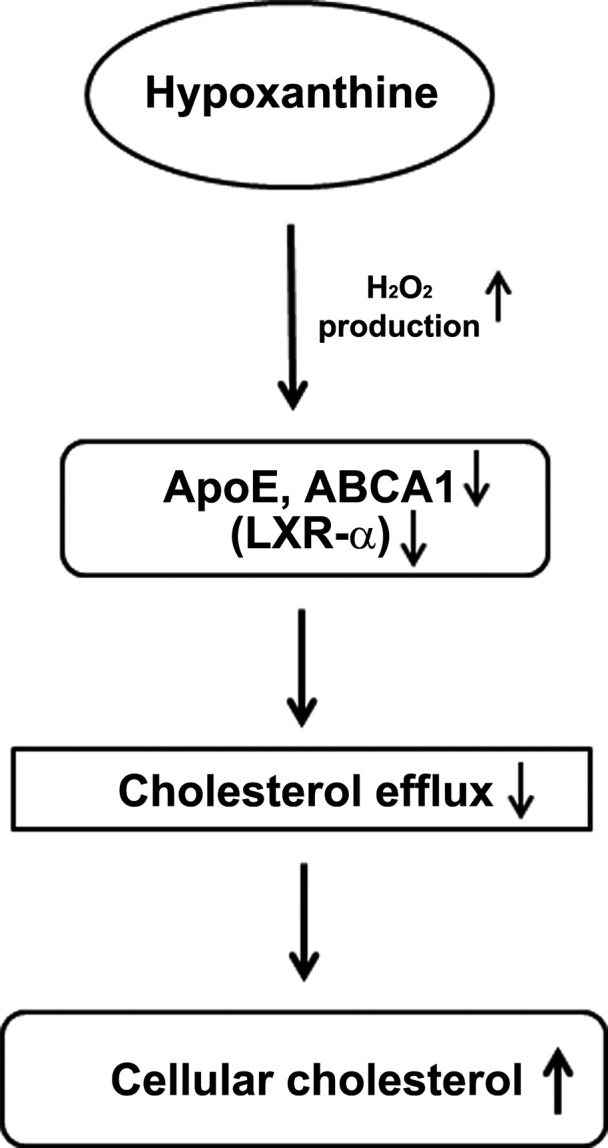
A diagram for cholesterol accumulation by hypoxanthine. In cells, hypoxanthine‐induced hydrogen peroxide production. The induction of hydrogen peroxide decreased cholesterol efflux by targeting ABCA1, ApoE and LXR‐α and led to accumulation of cellular cholesterol.

Taken together, our results demonstrate that hypoxanthine induces ROS generation and that ROS production leads to down‐regulation of the cholesterol transport genes, finally inducing cholesterol accumulation in hepatic cells (Fig. 7). These are accelerated in APOE deficiency. *In vivo*, hypoxanthine induces atherosclerosis in *Apoe‐*deficient mice. It is possible that hypoxanthine has a negative impact on lipid and atherosclerosis in cases where APOE is deficient such as for APOE4 carriers with dysfunctional APOE status. Eventually, hypoxanthine might accelerate atherosclerosis. In certain situations such as smoking, alcohol intake and haemodialysis, hypoxanthine levels can be elevated. Hypoxanthine level might be used to predict oxidative stress in cardiovascular diseases in all these situations.

## Conflict of interest

None declared.

## Supporting information


**Figure S1** Effect of hypoxanthine on HepG2 cell viability.
**Figure S2** Effect of hypoxanthine on serum uric acid level in C57BL/6 (WT) and Apoe KO mice.
**Table S1** Real‐time PCR primer sequences.
