# Supplementary material for: Hypoxanthine induces cholesterol accumulation and incites atherosclerosis in apolipoprotein E‐deficient mice and cells
Source: J Cell Mol Med. 2016 Jul 11;20(11):2160–72. doi: 10.1111/jcmm.12916 (PMC5082407; doi:10.1111/jcmm.12916)
Supplement: Supplementary file 1 — Figure S1 Effect of hypoxanthine on HepG2 cell viability. Figure S2 Effect of hypoxanthine on serum uric acid level in C57BL/6 (WT) and Apoe KO mice. Table S1 Real‐time PCR primer sequences. [file JCMM-20-2160-s001.doc]

**Supplementary Material**

**Supplementary Table 1: Real-time PCR primer sequences**

| **Primer** | **Sequence** |
| --- | --- |
| *APOE* (Forward) | 5′-TGGGTCGCTTTTGGGATTAC-3′ |
| *APOE* (Reverse) | 5′-TCAGCGCCCTCAGTTCCT-3′ |
| *ABCA1* (Forward) | 5′-GACATCGTGGCGTTTTTG G-3′ |
| *ABCA1* (Reverse) | 5′-CGAGATATGGTCCGGATTGC-3′ |
| *LDLR* (Forward) | 5′-AGTTGGCTGCGTTAATGTGACA-3′ |
| *LDLR* (Reverse) | 5′-TCTCTAGCCATGTTGCAGACTTTG-3′ |
| *HMGCR* (Forward) | 5′-GGACAGGATGCAGCACAGAA-3′ |
| *HMGCR* (Reverse) | 5′-GCATGGTGCAGCTGATATATAA ATCT-3′ |
| Human β-actin (Forward) | 5′-CTGTCCACCTTC CAGCAGATGT-3′ |
| Human β-actin (Reverse) | 5′-CGCAACTAAGTCATAGTCCGCC-3′ |

**
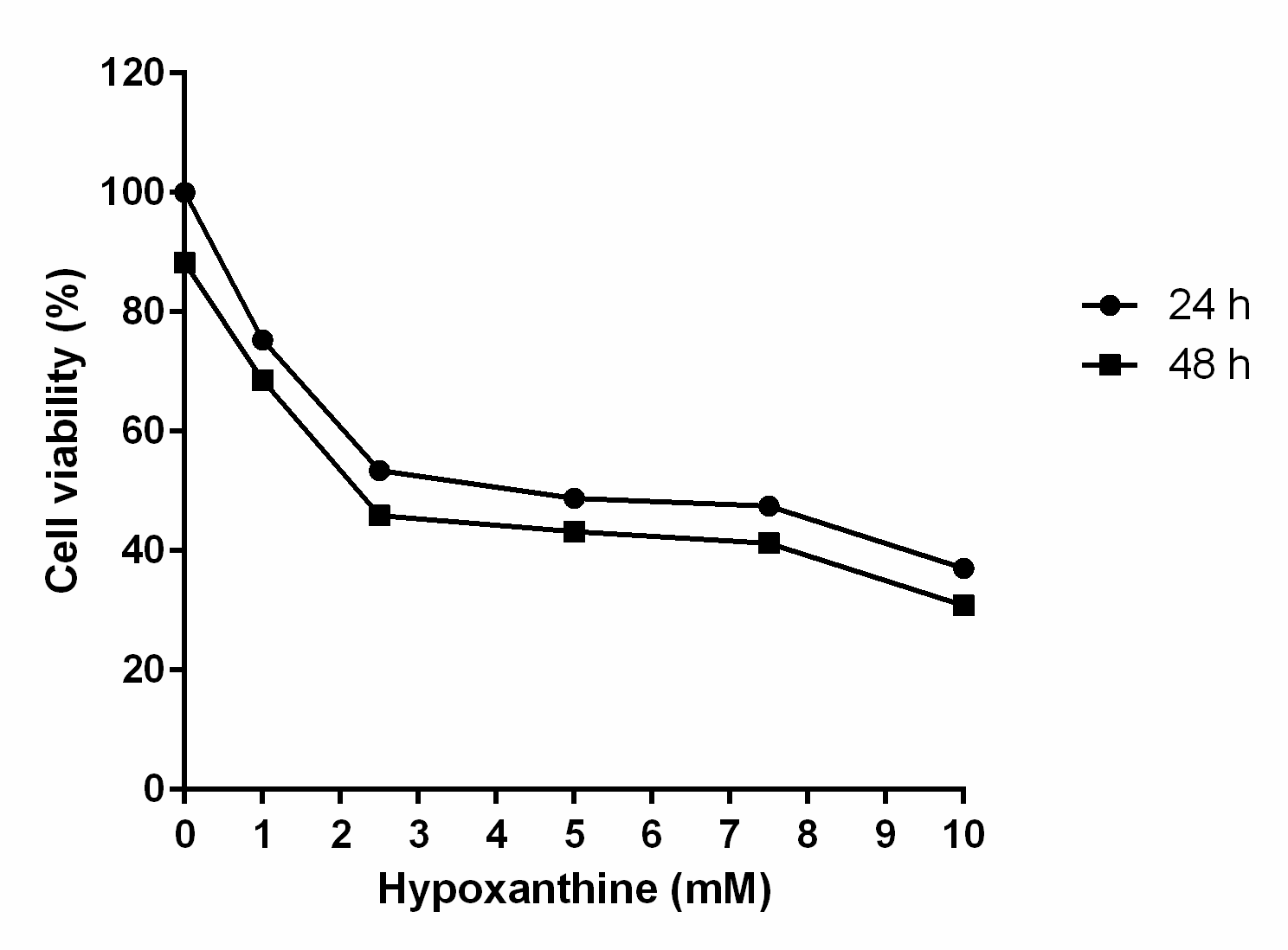
**

Supplementary Figure 1: Effect of hypoxanthine on HepG2 cell viability

Cell viability was assessed by using the MTT assay after HepG2 cells were treated with different concentrations of hypoxanthine (0, 1, 2.5, 5, 7.5, and 10 mM) for 24 and 48 h.


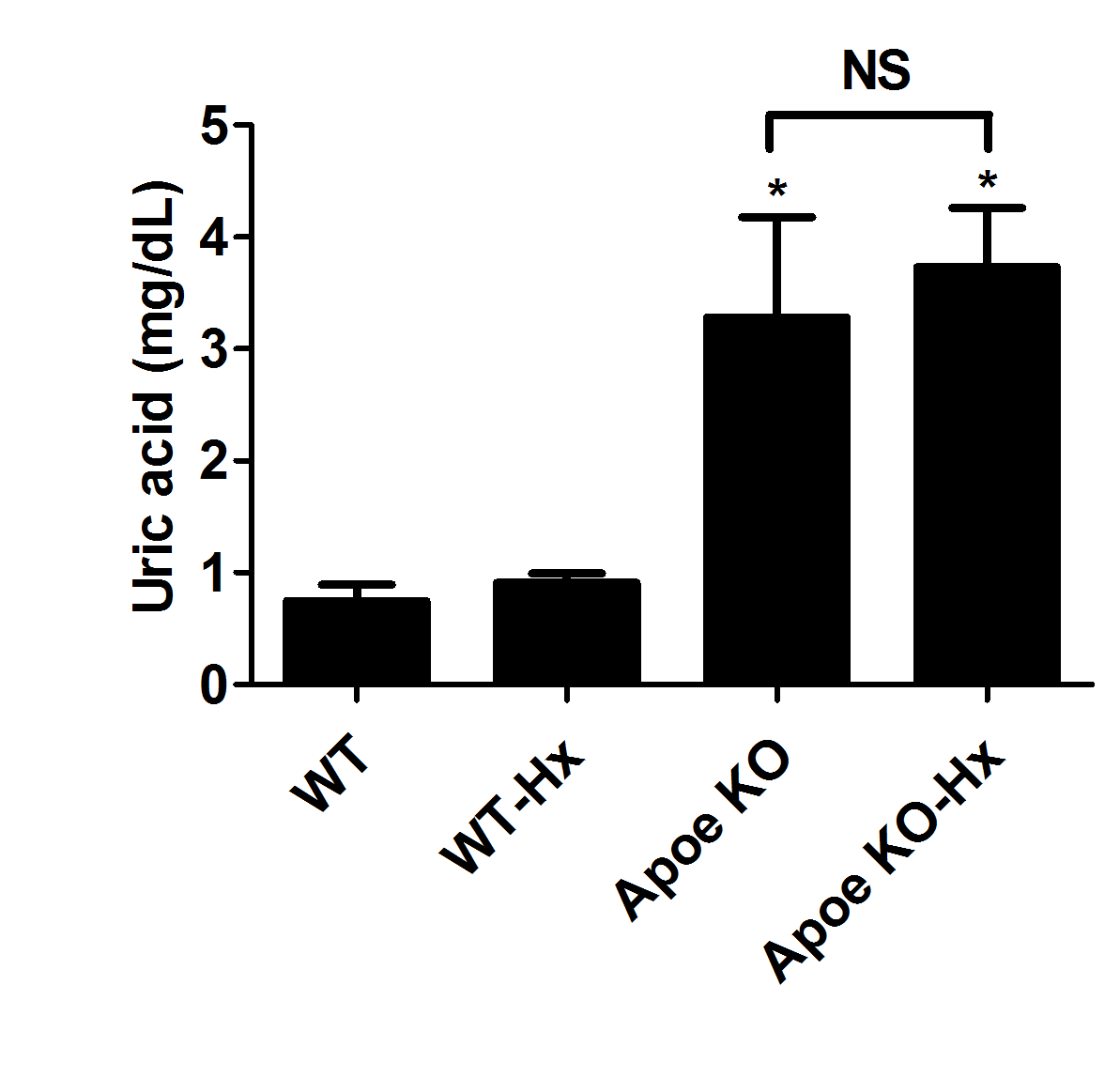


Supplementary Figure 2: Effect of Hypoxanthine on serum uric acid level in C57BL/6 (WT) and *Apoe* KO mice. Data are presented as mean ± SEM. **P* < 0.05 *versus* WT. Hx, hypoxanthine. NS, not significant.
